# Opportunistic Eye Screening Among First-Degree Relatives of Glaucoma Patients at a Suburban Tertiary Center in Malaysia

**DOI:** 10.7759/cureus.25772

**Published:** 2022-06-08

**Authors:** Radtthiga Chelvaraj, Maya Sapira Hanapi, Siti-Fairuz Mohd-Yusof, Khairy Shamel Sonny Teo, Liza Sharmini Ahmad Tajudin, Azhany Yaakub

**Affiliations:** 1 Department of Ophthalmology and Visual Science, School Of Medical Sciences, Universiti Sains Malaysia, Kubang Kerian, MYS; 2 Ophthalmology Clinic, Hospital USM, Universiti Sains Malaysia, Kubang Kerian, MYS

**Keywords:** opportunistic screening, pacg, joag, poag, glaucoma

## Abstract

Background and objective

The majority of glaucoma patients are asymptomatic and are usually diagnosed at an advanced stage of the disease. This study aimed to assess the outcomes of glaucoma screening among known first-degree relatives of primary glaucoma patients.

Materials and methods

This study involved primary angle-closure glaucoma (PACG), primary open-angle glaucoma (POAG), and juvenile open-angle glaucoma (JOAG) patients who attended the glaucoma clinic at the Hospital Universiti Sains Malaysia between January 2014 and December 2015. First-degree relatives of the patients underwent a preliminary eye-screening evaluation, including visual acuity (Snellen chart), intraocular pressure (IOP) measurement (air-puff tonometry), and non-mydriatic fundus photography. Patients with visual acuity worse than 6/12, IOP measuring more than 21 mmHg or a difference of more than 3 mmHg between the eyes, and a vertical cup-disc ratio (VCDR) of 0.7 or higher were given a comprehensive eye examination.

Results

Seventy indexed glaucoma patients were recognized, and 368 first-degree relatives were identified. Forty-five relatives underwent the preliminary screening. Of these, 29 showed normal findings (62%), one had corneal pathology (2%), and 16 (36%) underwent a complete eye examination after failing the initial screening. Among the indexed JOAG group, five relatives (11%) were diagnosed as having JOAG; two were treated medically, while the remaining three required surgical intervention.

Conclusion

Opportunistic glaucoma screening of high-risk groups, especially JOAG is a feasible and cost-effective way to detect early glaucoma and prevent irreversible blindness. However, improvement in our healthcare system, including the involvement of multicentre clinics in other states in screening initiatives, is required to promote and facilitate the response to screening opportunities.

## Introduction

Glaucoma is the leading cause of irreversible blindness worldwide and the second most common cause of blindness after cataracts [1]. Globally, glaucoma affects more than 60 million individuals, and an estimated 10% of those with glaucoma are blind [1,2]. Glaucoma involves the progressive loss of retinal ganglion cells (RGC) along with characteristic changes in the neuro-retinal rim tissue in the optic nerve head (ONH), which is accompanied by visual field (VF) constriction [3].

The Thessaloniki Eye Study conducted in Greece reported a high prevalence of undiagnosed glaucoma in the studied population (50%) [4]. This result is similar to that of the Blue Mountains Eye Study, conducted in Australia [5]. It is a matter of concern that most patients present for consultation at an advanced stage of glaucoma [6]. This is due to the fact that glaucoma is generally painless, and visual function may be preserved until the disease progresses to an advanced stage, as loss of the retinal nerve fiber layer precedes VF loss [3,6]. Thus, the majority of glaucoma patients are asymptomatic, which leads to late detection [3,6]. In addition, a general lack of awareness about this silent visual killer contributes to the lack of early glaucoma detection [6]. In addition to the increased risk of glaucoma among certain ethnic groups, including Hispanics and African-Americans, and the higher risk associated with aging, an increased risk of glaucoma is also well recognized among family members of known open-angle glaucoma patients [2,3].

Effective screening can reduce morbidity and improve the quality of life among glaucoma patients [7,8]. Population-based screening is impractical for glaucoma detection owing to the low sensitivity of intraocular pressure (IOP) measurement as a diagnostic screening tool and the difficulty of interpreting the appearance of the ONH [7,9]. Also, population screening has been found to be cost-ineffective because of the relatively low prevalence of glaucoma in the general population [9]. Finally, screening tools to diagnose glaucoma in the primary healthcare setting are not considered to be cost-effective in developing countries [6]. However, screening of populations with a high prevalence, such as those with a family history of glaucoma, has been found to be useful and screening tests for higher-risk groups have been found to have a positive predictive value [3,7,8]. Opportunistic glaucoma screening recruits patients who are at a high risk of developing glaucoma [8,9]. Several epidemiological studies have shown an increased risk of glaucoma in the family members of individuals with primary open-angle glaucoma (POAG) [9,10]. The Beaver Dam Eye Study, conducted in Beaver Dam, Wisconsin in the United States, found that close familial relation was a risk indicator for glaucoma [10]. The Baltimore Eye Survey reported that a family history of glaucoma was an important risk factor and that approximately 50% of glaucomas were hereditary in origin. In this study, family members of patients with POAG were shown to have a threefold increased risk of developing open-angle glaucoma [7]. Thus, an increased prevalence of glaucoma has been found in people whose first-degree relatives suffer from POAG [9,10]. Therefore, examination of the family members of patients suffering from primary glaucoma could be an effective way of identifying those at higher risk of having the disease and could ensure early detection and treatment to reduce glaucoma progression [9,11]. This study aimed to assess the feasibility and outcomes of glaucoma screening among first-degree relatives of primary glaucoma patients.

## Materials and methods

This cross-sectional study was exempted from ethical review by the Human Research Ethics Committee of Universiti Sains Malaysia as all the information obtained was anonymized. The study was carried out at the ophthalmology clinic of the Hospital Universiti Sains Malaysia between January 2014 and December 2015.

First-degree family members of known glaucoma patients with primary angle-closure glaucoma (PACG), POAG, and juvenile open-angle glaucoma (JOAG) were included in the study. Family members of probands with secondary open-angle glaucoma or pseudoexfoliation glaucoma were excluded from the study. Family members with incomplete data during screening were also excluded.

Three hundred and sixty-eight first-degree relatives of 70 indexed glaucoma probands were identified and divided into three groups: PACG, POAG, and JOAG. The pedigree of each patient was charted, and the contact details of first-degree relatives were obtained with permission from the probands. Only 151 of the 368 first-degree relatives identified were residents of Kelantan. The first-degree relatives were contacted and invited to undergo a screening test. However, only 45 relatives attended the screening. The examinations performed by paramedics during the eye screening included visual acuity (Snellen chart), IOP measurement with air-puff tonometry, and ONH photo using non-mydriatic funduscopy. The cut-off points for referral for comprehensive examination were vision worse than 6/12, IOP more than 21 mmHg or a difference greater than 3 mmHg between the eyes, and a vertical cup-disc ratio (VCDR) of 0.7 or higher.

Individuals who met these criteria were classified as suspicious of having glaucoma and underwent a detailed examination to obtain a definitive diagnosis. Relatives who were diagnosed with glaucoma were managed with anti-glaucoma medications and surgical treatment. Other ocular abnormalities were referred to a general eye clinic for further evaluation. Information about glaucoma was also provided during the screening.

All data were entered and statistical analyses were performed using IBM SPSS Statistics version 21 (IBM, Armonk, NY).

## Results

**Figure 1:**
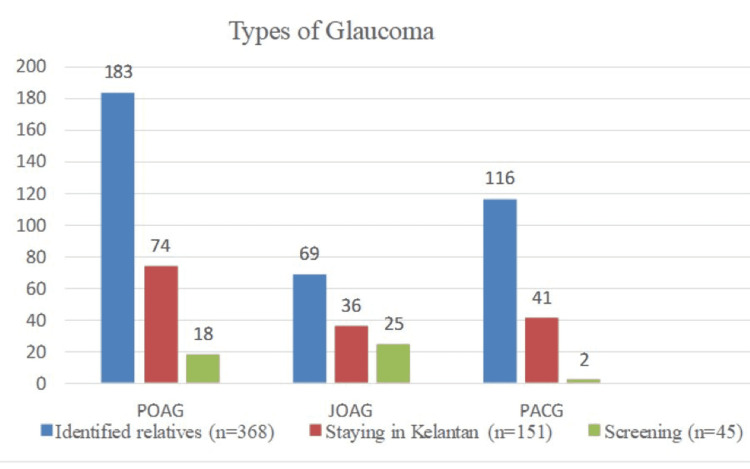
Number of identified glaucoma relatives, number of relatives who are staying in Kelantan, and the number of relatives who underwent screening according to the type of glaucoma POAG: primary open-angle glaucoma; JOAG: juvenile open-angle glaucoma; PACG: primary angle-closure glaucoma

While a total of 151 (41%) relatives resided in the Kelantan state, only 45 of these relatives attended the glaucoma screening: 20 (44%) relatives with a family history of POAG, 23 (51%) with a family history of JOAG, and two (4%) with a family history of PACG. The majority of the probands were Malays (95%). Figure 1 shows the number of identified glaucoma relatives, the number of relatives residing in Kelantan, and the number of relatives who underwent screening.

Of the 45 first-degree relatives who were screened, 16 (36%) were classified as suspicious for glaucoma, while 28 (62%) had normal findings, and one (2%) had a cornea problem. The screening outcomes are provided in Table 1.

**Table 1:** Outcomes of screening among relatives according to the type of glaucoma POAG: primary open-angle glaucoma; JOAG: juvenile open-angle glaucoma; PACG: primary angle-closure glaucoma

	Group of relatives			Total (n=45)
	POAG (n=18)	JOAG (n=25)	PACG (n=2)	
Glaucoma suspect	3	12	1	16
Confirmed as glaucoma	(0)	(5)	(0)	(5)
Other problem	0	1	0	1
Normal and discharged	15	12	1	28

Among the indexed JOAG group, five relatives (11%) were diagnosed with JOAG; two of them were treated with topical eye drops alone, and three required a combination of topical eye drops and surgical intervention, as shown in Table 2. No cases of POAG or PACG were diagnosed in the glaucoma-suspect group.

**Table 2:** Treatment modalities received by newly diagnosed and confirmed glaucoma cases detected by screening

Treatments	Number of cases (n=5)
Topical eye drops	2
Topical eye drops + trabeculectomy	1
Topical eye drops + glaucoma drainage device	2

## Discussion

In this study, we assessed the prevalence of glaucoma among the first-degree relatives of PACG, POAG, and JOAG patients. We screened the first-degree relatives of primary glaucoma patients to explore the feasibility of identifying individuals at risk of glaucoma based on their family’s medical history.

Several previous studies have shown that primary glaucoma is more likely to affect persons with a family history of the disease, and a positive family history has been assumed to be associated with a significant risk of glaucoma [9,10]. One study found that a positive family history of glaucoma was associated with open-angle glaucoma, and the strongest association was found among siblings of an affected person [8]. Our study detected five cases of glaucoma in the siblings of known JOAG patients. Our findings are similar to those of studies conducted in India and the United States [9,10]. Studies have shown that PACG is common among people of Chinese ethnicity [12]. Hence, fewer PACG patients were encountered in our study with their probands as the majority of the relatives were Malay. Based on our study findings, opportunistic eye screening for primary glaucoma targeting individuals with a positive family history may be worthwhile, although this would cover only a small percentage of the population. Along with the high probability of detecting glaucoma among the relatives of primary glaucoma patients, opportunistic screening can also save on costs and labor.

The glaucoma-screening tools used in our study were selected based on their feasibility in our local setting. The instruments were both user-friendly for our paramedics and non-invasive, which helped to eliminate anxiety among the individuals who were screened. We measured IOP using air-puff tonometry, as it is relatively quick and does not require any contact, thus reducing anxiety in the person being screened. In addition, the non-mydriatic fundus camera is a desirable screening tool for glaucoma, as it is widely available at primary healthcare clinics in the country and thus contributes to the accessibility of glaucoma-screening programs. Other tools that can be used to assess risk factors for the development and progression of glaucoma include optical coherence tomography (OCT) for structural assessment, the Humphrey VF analyzer to assess VF defects, and the ultrasonic pachymeter to assess the central cornea thickness [13].

This study was conducted in Kelantan state, one of Malaysia’s most economically underdeveloped areas, located in the north-eastern region of Peninsular Malaysia. According to the Department of Statistics in Malaysia, the gross domestic product (GDP) per capita in Kelantan was Malaysian ringgit (RM) 12,075 in 2015, while the national per capita income was RM 37,104. Thus, it is not surprising that the majority of first-degree relatives were living and working outside the Kelantan state. This explains the poor attendance rate for this screening program. Another possible cause for the low attendance rate for the screening program in this study was the lack of awareness about glaucoma among relatives. Several previous studies have highlighted that a family history of glaucoma and level of education were determinants of glaucoma awareness [10,14]. This targeted population screening could serve as a basis for conducting more effective screening programs in the future. In this study, five members of 41 screened families were diagnosed with JOAG and received appropriate treatment. There would have been a possibility of identifying more relatives with glaucoma or who are at a higher risk of developing glaucoma if more relatives had attended the screening.

The study was limited by the fact that it was performed at a single center with a significantly low rate of participation by the targeted subjects. Effective glaucoma screening among relatives of JOAG patients, in particular, could be enhanced by setting up user-friendly screening centers across the country at the primary healthcare level and by raising glaucoma awareness among relatives.

## Conclusions

In our pilot study, glaucoma screening among first-degree relatives showed tangible benefits, especially among the JOAG group. However, it was inconclusive for the POAG and PACG groups. Opportunistic screening of high-risk groups and collaboration between multiple centers across the country would improve the feasibility of early glaucoma detection. More efforts should be undertaken to educate the community about glaucoma, which may in turn improve participation in glaucoma-screening programs.
